# Declarations of conflicts of interest from the editors of the *Journal of Global Health*

**DOI:** 10.7189/jogh.08.010102

**Published:** 2018-06

**Authors:** Ana Marušić, Igor Rudan, Harry Campbell

As the editors of the *Journal of Global Health*, we are committed to ensuring that all processes during the publication of articles in our journal have the same level of transparency and responsibility. As we expect from our authors and reviewers to declare their possible conflicts of interest in relation to the manuscript under consideration for publication in the journal, we also need to declare our commitments and relations that may influence the editorial review process. When one of the decision-making editors submits an article to the *Journal*, it is reviewed independently from the editor, who has no access to or influence on the review process and editorial decision-making. This is always indicated in the Competing Interests declaration at the end of the published article.

As the next step in increasing the transparency of the editorial conflicts of interest [1] and in order to be compliant with the International Committee of Medical Journal Editors (ICMJE) Recommendations for the Conduct, Reporting, Editing, and Publication of Scholarly work in Medical Journals [2], we declare the following conflicts of interest for the current year:

## Professor Ana Marušić, MD, MS, PhD

**Personal and organizational**
Prof. Marušić is fully employed by the University of Split School of Medicine, where she holds a tenured position as Professor and Chair of the Department of Research in Biomedicine and Health. She receives funding support for research from the Croatian Science Foundation and the European Commission under the Horizon 2020 programme.

**Journal**
Prof. Marušić holds a position as the Editor in Chief. She occasionally reimburses small travel expenses related to Journal’s work.

**Unpaid positions**
Honorary Professor, College of Medicine and Veterinary Medicine, University of Edinburgh, Edinburgh, Scotland, UK
Visiting Professor, School of Medicine, University of Sao Paulo, Sao Paulo, Brazil
President, European Association of Science Editors (until June 2018)
Steering Group, EQUATOR Network
Co-Chair, Cochrane Scientific Committee
Research Coordinator, Cochrane Croatia
Advisory Board Member, the *Balkan Medical Journal*

## Professor Igor Rudan, MD, DSc, PhD, MPH, FRSE

**Personal and organizational**
Prof. Rudan is employed by the University of Edinburgh, where he holds a position as Professor and Co-Director of the Centre for Global Health Research. He is also co-Director of the WHO Collaborating Centre in Population Health Research and Training. He currently receives research funding support from the Gates Foundation.

**Journal**
Prof. Rudan holds a position as the co-Editor in Chief. He occasionally reimburses small expenses related to Journal’s work, including travel and consumables.

**Paid positions**
Prof. Rudan has numerous technical advisor appointments to WHO, UNICEF and the World Bank.

## Professor Harry Campbell, MD, FRCPE, FFPH, FRSE

**Personal and organizational**
Prof. Campbell is employed by the University of Edinburgh, where he holds a position as Professor and Co-Director of the Centre for Global Health Research. He is also co-Director of the WHO Collaborating Centre in Population Health Research and Training and co-Director of the NIHR Global Respiratory Health Unit. He currently receives research funding support from the European Commission (Innovative Medicines Initiative), WHO, UK NIHR, Cancer Research UK, UK MRC, Sanofi (for work on specific GI and respiratory infections) and the Gates Foundation.

**Journal**
Prof. Rudan holds a position as the co-Editor in Chief. He occasionally reimburses small expenses related to Journal’s work, including travel and consumables.

**Unpaid positions**
Member, MRC Africa Research Excellence Fund (AREF), College of Experts, since 2016
Member, WHO PIP burden of disease advisory group, since 2015
Member of international Scientific Advisory Group, INDEPTH network, since 2014
Member of Expert Advisory Group for “The Making of Genomic Medicine Wellcome Trust Programme; University of Edinburgh), since 2013
Member, Public Health, Health Services Research and Primary Care (A; subpanel 2) Research Excellence Framework (REF), 2018-2021
Numerous technical advisor appointments to WHO in the past 5 years
